# Supplementary material for: Isobolographic Analysis Demonstrates the Additive and Synergistic Effects of Gemcitabine Combined with Fucoidan in Uterine Sarcomas and Carcinosarcoma Cells
Source: Cancers (Basel). 2019 Dec 31;12(1):107. doi: 10.3390/cancers12010107 (PMC7017062; doi:10.3390/cancers12010107)
Supplement: Supplementary file 1 [file cancers-12-00107-s001.pdf]

cancers

MDPI

Supplementary Materials: Isobolographic Analysis Demonstrates the Additive and Synergistic Effects of Gemcitabine Combined with Fucoidan in Uterine Sarcomas and Carcinosarcoma Cells

Suppl. Table S1. Anti-proliferative effects of gemcitabine (GEM) and fucoidan (FUK) administered singly in cancer cell lines ESS-1, SKUT-1, SKUT-1B and MES-SA, as measured *in vitro* by the MTT assay.

Cell line	Drug	IC_{50} (µg/ml)	*n*
SKUT-1	GEM	31.173 ± 5.608	30
SKUT-1	FUK	0.966 ± 0.541	24
SKUT-1B	GEM	25.243 ± 7.522	24
SKUT-1B	FUK	3.348 ± 2.317	24
ESS-1	GEM	13.875 ± 2.207	24
ESS-1	FUK	0.848 ± 1.129	30
MES-SA	GEM	72.482 ± 26.070	30
MES-SA	FUK	N.D.	

Median inhibitory concentrations (IC_{50} values in µg/ml ± S.E.M.) of GEM and FUK were determined experimentally in various cancer cell lines. *n* – total number of items used at those concentrations whose expected anti-proliferative effects ranged between 4 and 6 probits (16% and 84%). The results for fucoidan (FUK) have been previously published by our team [10].

Suppl. Table S2. Type I isobolographic analysis of interactions (for non-parallel dose-response effects) between gemcitabine (GEM) and fucoidan (FUK) in three cancer cell lines ESS-1, SKUT-1 and SKUT-1B, as measured *in vitro* by the MTT assay.

Cell line	Combination	$IC_{50\,mix}$ (µg/ml)	n_{mix}	$^{L}IC_{50\,add}$ (µg/ml)	n_{add}	$^{U}IC_{50\,add}$ (µg/ml)	n_{add}
ESS-1	GEM+FUK	0.101 ± 0.052	24	2.508 ± 2.164	50	12.215 ± 2.270	50
SKUT-1	GEM+FUK	9.016 ± 2.493	30	4.850 ± 6.082	50	27.290 ± 7.093	50
SKUT-1B	GEM+FUK	4.713 ± 1.714	30	10.728 ± 4.748	44	17.866 ± 6.619	44

Median inhibitory concentrations (IC_{50} values in µg/ml ± S.E.M.) for the mixture of GEM+FUK were determined both, experimentally ($IC_{50\,mix}$) and theoretically calculated ($IC_{50\,add}$) from the equations of additivity for a 50% inhibition of cell proliferation in three cancer cell lines ESS-1, SKUT-1 and SKUT-1B. n_{mix} – total number of items used at those concentrations whose expected anti-proliferative effects ranged between 16% and 84% (i.e., 4 and 6 probits) for the experimental mixture; n_{add} – total number of animals calculated for the additive mixture of the drugs examined ($n_{add} = n\_{GEM} + n\_{FUK} - 4$); $^{L}IC_{50\,add}$

and $^{U}IC_{50\ add}$ – are IC_{50} values (in μg/ml ± S.E.M.) calculated from the equations for the lower and upper isoboles of additivity, respectively. The unpaired Student's *t*-test was used to statistically analyze the data.

Suppl. Table S3. Statistical significance of differences in percents of cells in each cell cycle phases among cells treated with gemcitabine or mixture (gemcitabine and fucoidan) and control (no treatment). The results were analyzed by one-way ANOVA test, Tukey's Multiple Comparison Post-test, $p < 0.05$ was considered as statistically significant. The results of experiment are presented on figure 7.

<table>
<tr><th rowspan="2">Cell line</th><th colspan="4">Cell cycle phase</th></tr>
<tr><th>pre G1</th><th>G0/G1</th><th>S</th><th>G2</th></tr>
<tr><td colspan="5">SK-UT-1</td></tr>
<tr><td>control vs GEM</td><td>p<0.01</td><td>NS</td><td>p<0.05</td><td>NS</td></tr>
<tr><td>control vs 0.5 IC50 FUK+GEM</td><td>NS</td><td>NS</td><td>p<0.01</td><td rowspan="2">p<0.05</td></tr>
<tr><td>GEM vs 0.5 IC50 FUK+GEM</td><td>p<0.05</td><td>NS</td><td>p<0.001</td></tr>
<tr><td colspan="5">SK-UT-1B</td></tr>
<tr><td>control vs GEM</td><td>NS</td><td>NS</td><td>p<0.05</td><td rowspan="3">p<0.001</td></tr>
<tr><td>control vs 0.5 IC50 FUK+GEM</td><td rowspan="2">p<0.001</td><td rowspan="2">p<0.001</td><td rowspan="2">p<0.001</td></tr>
<tr><td>GEM vs 0.5 IC50 FUK+GEM</td></tr>
<tr><td colspan="5">ESS-1</td></tr>
<tr><td>control vs GEM</td><td>p<0.001</td><td>p<0.01</td><td>p<0.05</td><td rowspan="2">p<0.001</td></tr>
<tr><td>control vs 0.05 IC50 FUK+GEM</td><td>NS</td><td>NS</td><td>p<0.01</td></tr>
<tr><td>GEM vs 0.05 IC50 FUK+GEM</td><td>NS</td><td>NS</td><td>NS</td><td>NS</td></tr>
</table>

© 2020 by the authors. Submitted for possible open access publication under the terms and conditions of the Creative Commons Attribution (CC BY) license (http://creativecommons.org/licenses/by/4.0/).
